# Differences in the microRNAs Levels of Raw Milk from Dairy Cattle Raised under Extensive or Intensive Production Systems

**DOI:** 10.3390/vetsci9120661

**Published:** 2022-11-27

**Authors:** Loubna Abou el qassim, Jaime Alonso, Ke Zhao, Sandrine Le Guillou, Jorge Diez, Fernando Vicente, Manuel Fernández-Sanjurjo, Eduardo Iglesias-Gutiérrez, Leluo Guan, Luis J. Royo

**Affiliations:** 1 Servicio Regional de Investigación y Desarrollo Agroalimentario (SERIDA), Carretera AS-267, PK. 19, 33300 Villaviciosa, Spain; 2 Centro de Inteligencia Artificial, Universidad de Oviedo, Campus de Viesques, 33271 Gijón, Spain; 3 Institute of Food Science, Zhejiang Academy of Agricultural Sciences, Hangzhou 310004, China; 4 INRAE, AgroParisTech, GABI, Université Paris-Saclay, 78350 Jouy-en-Josas, France; 5 Department of Functional Biology, Physiology, University of Oviedo, 33006 Oviedo, Spain; 6 Instituto de Investigación Sanitaria del Principado de Asturias (ISPA), 33011 Oviedo, Spain; 7 Department of Agricultural, Food and Nutritional Science, University of Alberta, Edmonton, AB T6G 2R3, Canada

**Keywords:** milk, miRNA, dairy production systems, biomarker

## Abstract

**Simple Summary:**

Extensive animal production systems are generally considered more sustainable and beneficial for the environment and for maintaining rural populations. However, there is no defined concept of what extensive milk production is. It is assumed to be a kind of production based on pasture and forage, with animals spending part of their daytime free and with a low stocking density. In order to increase consumer confidence in this type of product, we have studied markers based on molecules naturally present in the milk that allow us to differentiate the production system in which the cows that have produced the milk have come from. In addition, we are attempting to determine whether the milk production system can have benefits for consumer health.

**Abstract:**

Studying microRNA (miRNAs) in certain agri-food products is attractive because (1) they have potential as biomarkers that may allow traceability and authentication of such products; and (2) they may reveal insights into the products’ functional potential. The present study evaluated differences in miRNAs levels in fat and cellular fractions of tank milk collected from commercial farms which employ extensive or intensive dairy production systems. We first sequenced miRNAs in three milk samples from each production system, and then validated miRNAs whose levels in the cellular and fat fraction differed significantly between the two production systems. To accomplish this, we used quantitative PCR with both fractions of tank milk samples from another 20 commercial farms. Differences in miRNAs were identified in fat fractions: overall levels of miRNAs, and, specifically, the levels of *bta-mir-215,* were higher in intensive systems than in extensive systems. Bovine mRNA targets for *bta-miR-215* and their pathway analysis were performed. While the causes of these miRNAs differences remain to be elucidated, our results suggest that the type of production system could affect miRNAs levels and potential functionality of agri-food products of animal origin.

## 1. Introduction

Consumers’ growing concern about food characteristics has contributed to the creation of a new concept of quality, particularly for animal products. This concept includes traditional attributes related to nutritional value, flavor, aroma, and color, together with new indicators related to ethical aspects, such as animal welfare and environmental impact of the production system [1]. Consumers assume that products from cows raised under pasture and/or grazing are more natural and better for meeting animal welfare demands than those from the cows raised under the cereal-rich diets typical of intensive production systems. Intensive systems are considered less sustainable than production based on pastures, particularly because they have a larger ecological footprint and because they divert cereals from human consumption. These considerations highlight the need to compare whether agri-food products obtained from one production system or the other may have advantages, so that consumers can make fully informed purchasing decisions [2].

Milk is a complex secretory product and a source of nutrients for children and adults. Milk also contains numerous biomolecules, including microRNAs (miRNAs), a group of non-coding RNAs that bind to specific regions within messenger RNA to regulate gene expression post-transcriptionally [3]. In vitro models [4] have suggested that exogenous miRNAs, such as bovine milk miRNAs, may influence the health of humans due to their resistance to stomach digestion, and that they may affect cellular function. Nevertheless, the potential bioactivity of dietary miRNAs is still under study, and several aspects remain poorly understood, including the dose needed to produce biological effects [5]. The miRNA level of milk depends on genetic factors, such as the animal’s breed and physiology [6,7], as well as environmental factors, such as the animal’s lifestyle, pathological state, and diet [8,9,10,11]. Furthermore, the miRNA level depends on the milk fractions, milk fat, whey, and cells [12]. The miRNAs found in milk are highly resistant to acidic pH and to enzymes such as RNases [13], which implies that they are very stable and resistant to industrial treatments [13,14,15]. For these reasons, miRNAs have been proposed as biomarkers of quality control in dairy products, and they have been used to control for fraud in the labeling of milk powder [14].

Changes in diet have been shown to affect the expression of genes in the mammary gland [16,17]. Considering that miRNAs are essential regulators of gene expression, miRNA profiles in milk may also change with diet. However, few studies have investigated the effect of diet on miRNA profiles in bovine milk [18], such as through comparisons of the quite different diets in intensive or extensive production systems. Therefore, our objective was to evaluate differences in miRNA levels of bovine milk produced under extensive or intensive systems. Characterizing production systems as intensive or extensive is not always straightforward, given the lack of regulatory definitions and the complexity of the factors involved [19]. In the present study, we defined intensive systems as those without grazing and with high amounts of maize silage and concentrate in the diet. We defined extensive systems as those with grazing and a diet with a low amount of concentrate and no maize silage. Our work was carried out in Asturias, north of Spain, where the edapho-climatic conditions permit the coexistence of different milk production systems, varying from intensive systems, with animals fed indoors, to extensive pastured-based systems. This results in a fairly wide range of production systems covering all management possibilities [20]. Furthermore, we also explored the use of miRNA markers for the characterization and traceability of agri-food products. We used bioinformatics to predict what target genes may be regulated by miRNAs whose levels differ between the two production systems, in an effort to examine how certain miRNAs may reveal the functional properties of agri-food products.

## 2. Materials and Methods

### 2.1. Sample Collection and Preparation

In order to maximize potential differences in milk composition between intensive and extensive production systems, for sequencing, we sampled six farms at the end of one spring season (May 2016). On three farms (extensive production), animals grazed for at least for 12 h each day and ate a diet based on fresh grass with a small amount of concentrates. On three farms (intensive production), animals did not graze, instead eating a diet based on conserved feed and concentrates. Farm characteristics and details of the diets are described in Table 1. All animals on all farms were Holstein cows.

For validation by RT-qPCR, twenty farms were sampled during autumn 2017 and spring 2018. Ten dairy farms applied an extensive system in which animals grazed and consumed a diet based on fresh grass, without maize silage and with a low amount of concentrates (4–8 kg/animal/day), while the other ten applied an intensive system in which animals were housed and ate a diet consisting of a high dry matter intake of maize silage (16–30 kg/animal/day) and a high amount of concentrate (>10 kg/animal/day) (Table 2).

From each type of farm, bulk tank milk was sampled after an even number of milkings (to avoid differences due to afternoon and morning milk composition). Samples were maintained at 4 °C and immediately transported to the laboratory for processing. Milk was well mixed, and 50 mL of each sample was centrifuged at 1900× *g* for 20 min. The fat in the upper phase was transferred to a new 50-mL RNase-free tube. Then, 7.5 mL of QIAzol lysis reagent (Qiagen, Crawley, UK) was added, and the emulsion was vigorously mixed until the fat was well dispersed. The pellet (cellular fraction) from the initial centrifugation was washed twice with phosphate-buffered saline, then homogenized with 1 mL of QIAzol lysis reagent. All samples were stored at −80 °C until RNA extraction.

### 2.2. RNA Isolation

For each sample, total RNA was extracted from 2 mL of milk fat in QIAzol lysis reagent, and from 1 mL of cell pellet resuspended in QIAzol lysis reagent. RNA was extracted using the miRVana miRNA isolation kit (Applied Biosystems, Foster City, CA, USA) following the manufacturer’s instructions, then stored at −80 °C. The concentration and integrity of RNA (RIN: RNA Integrity Number) for sequencing was further determined on an Agilent 2100 Bioanalyzer using an RNA 6000 Pico kit (both from Agilent Technologies, Santa Clara, CA, USA).

### 2.3. Search for miRNAs Candidates from Sequencing

#### 2.3.1. RNA Sequencing

In order to identify miRNAs differing between the two production systems, 12 libraries corresponding to the fat or cellular fractions of milk from six samples of bulk tank milk (three per production system) were prepared and sequenced using the Illumina platform (Illumina, San Diego, CA, USA) as 50 bp single reads. The raw sequence data were processed for quality control, and low quality reads were removed from raw data using CASAVA 1.8 based on chastity. Then, adaptors were trimmed, and sequences with read lengths between 15 to 40 nt were mapped to the bovine genome (bostau 7) and the miRNA database (miRBase, release version 21) in order to identify the known miRNAs. Expression levels of each miRNA were estimated based on the frequency of reads, and results were normalized to the number of reads per million (RPM) using the following formula: RPM = (specific miRNA reads number/total mapped miRNA reads per library) × 10^6^.

#### 2.3.2. Identification of Reference miRNAs for qPCR Normalization

In order to select the miRNAs to be used as candidates for normalizers in RT-qPCR, we chose those miRNAs with more stable expression among samples for each milk fraction, that is, miRNAs with the smallest coefficient of variation (CV = standard deviation/mean).

#### 2.3.3. Identification of miRNAs Whose Levels Differed between Production Systems

In order to identify those miRNAs whose levels differ between production systems, the results from miRNA sequencing were analyzed using three statistical tests. One test was the ratio of the difference between the means to the sum of the standard deviations between the two production systems: value = |(mean1–mean2)|/(standard deviation1 + standard deviation2) (strict Cohen’s d). This ratio indicates how many sums of deviations fit between the means; the higher the ratio is, the greater the difference between the means [21]. Another test was Student’s t test, for which lower t values indicated greater differences between the means for the two production systems [22]. The third test was the absolute value of the correlation coefficient; the higher this value was, the greater the difference between the two systems [23]. Afterward, to choose the miRNAs whose levels differed between production systems, they were ranked first according to each statistical test, and then according to the average of the classification of the three tests.

### 2.4. Validation of Candidate miRNAs Using RT-qPCR

#### 2.4.1. RT-qPCR Analysis

A subset of the miRNAs which were determined to differ significantly between the two production systems identified in Section 3.2 were validated using quantitative RT-PCR and milk samples from twenty dairy farms featuring extensive or intensive production systems (Table 2).

Total RNA was used for cDNA synthesis using the TaqMan Advanced miRNA cDNA Synthesis Kit (Thermo Fisher Scientific, Waltham, MA, USA), and the resulting cDNA was stored at −20 ℃ until use. Levels of miRNAs were determined using quantitative RT-PCR (TaqMan Advanced miRNA Assays; ThermoFisher Scientific, Waltham, MA, USA) in a StepOne thermocycler (Applied Biosystems, Foster City, CA, USA). The final reaction solution contained 10 μL of 2 × TaqMan Fast Advanced Master mix (ThermoFisher Scientific, Waltham, MA, USA), 1 μL of 20× TaqMan Advanced miRNA Assay (ThermoFisher Scientific, Waltham, MA, USA), 4 μL of RNase free water, and 5 μL of cDNA (diluted 1:10). The thermocycling program was set at 95 °C for 20 s, followed by 40 cycles at 95 °C for 1 s and 60 °C for 20 s. All PCR reactions were performed in duplicate, and a maximum of 0.5 threshold cycles were permitted between duplicates.

#### 2.4.2. Selection of Stable Reference miRNAs. GeNorm Analysis

Normalization is an essential component of a reliable qPCR assay. geNorm [24] is one of the most popular algorithms to find stable reference genes from a set of tested candidate reference genes in a given experimental condition. We used geNorm to find the optimal number and choice of reference genes for normalization, using miRNAs identified in Section 3.2 in 22 tank milk samples, representing the experimental variation of the dairy production systems existing in the area of study [25].

#### 2.4.3. miRNAs Levels Normalization and Estimation

Levels of miRNA were normalized based on the geometric mean of the selected reference miRNAs selected by geNorm, estimated using QBase+ 3.1 software, Biogazelle (Gent, Belgium) [26] and expressed in base log10. Unless otherwise noted, results were reported as mean ± standard deviation. Mean miRNAs levels between extensive and intensive production systems were compared using Student’s t test in R-Commander 2.7-1. Significance was defined as *p* < 0.05.

### 2.5. Prediction and Functional Analysis of Genes Targeted by miRNAs

The Target Scan 7.2 bioinformatics tool [27] was used to predict bovine mRNA targets of candidate miRNAs. A pathway analysis of targeted genes was performed using Panther bioinformatics tool version 16.0 (http://www.pantherdb.org/ accessed on 8 June 2021) based on Gene Ontology classification [28].

## 3. Results

### 3.1. miRNAs Levels in Fat and Cellular Fractions of Milk

The total mean RNA concentration was 192.4 ± 37.4 ng/µL in milk fat and 30.6 ± 15.8 ng/µL (mean ± SD) in milk cells. The RIN value was 2.6 ± 0.15 for RNA from milk fat, and 6.5 ± 0.3 for RNA from milk cells.

The six libraries from the cellular fraction of milk yielded a mean of 24,017,290.17 reads, significantly more than the 6,964,122.33 reads from the six libraries from the fat fraction (*p* = 0.004, Table 3). Almost half of the reads came from small RNAs, of which the most abundant were transfer RNAs (tRNAs) in the cellular fraction and non-coding RNAs (ncRNAs) in the fat fraction. Significant differences were found between the fat and cellular fractions for a percentage of all small RNAs, except for small nuclear RNAs (snRNAs) and miRNAs. Ribosomal RNAs (rRNAs), small nucleolar RNAs (snoRNAs), and ncRNAs were more abundant in the fat fraction than in the cellular fraction, while the converse was true for tRNAs. The levels of miRNAs in the fat fraction differed significantly between extensive and intensive systems (*p* = 0.040, Table 4). Intensive production was associated with higher miRNAs levels. 

We identified 518 known miRNAs in the cellular fraction of milk and 477 in the fat fraction. Most of these miRNAs (454) were present in both fractions (Table S1).

### 3.2. Validation of miRNAs Whose Levels Differed between Intensive and Extensive Production

The first 10 miRNAs in the fat fraction whose levels differed between the two production systems are ranked in Table 5, and those in the cellular fraction are ranked in Table 6. Levels of the first five miRNAs from each fraction were validated using quantitative RT-PCR and milk samples from another 20 farms. The following miRNAs in the fat fraction were subjected to validation: bta-miR-215, bta-miR-369-3p, bta-miR-6520, bta-miR-7863, and bta-miR-133a. The following miRNAs in the cellular fraction were subjected to validation: bta-miR-574, bta-miR-3432a, bta-miR-2285e, bta-miR-197, and bta-miR-2284y.

Six miRNAs in each milk fraction were chosen as candidates for normalization (Table 7). Following GeNorm, normalization of levels in the fat fraction was optimal using the geometric mean of bta-miR-151-3p and bta-miR-30a-5p. In the case of cellular fraction, GeNorm recommended the use of the geometric mean of the three most stable miRNAs (bta-miR-103, bta-miR-107, bta-miR-28).

**Figure 1:**
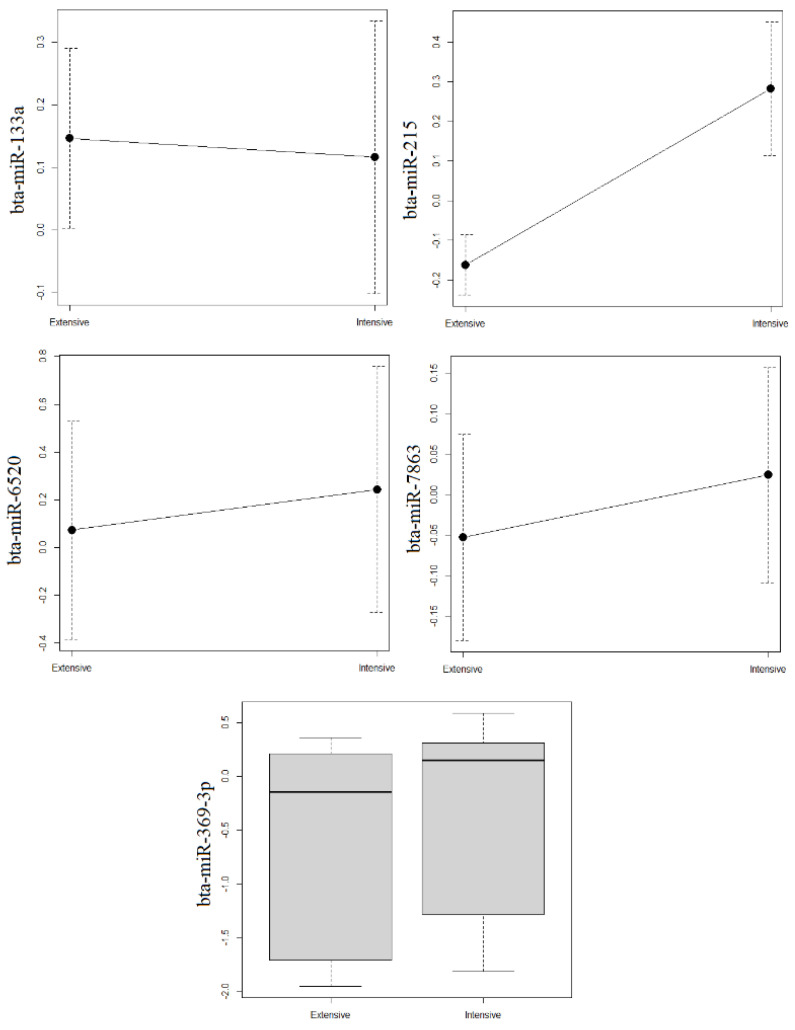
Average levels of miRNAs estimated by qRT-PCR in fat fractions of milk from extensive and intensive farms. On the X axis, production systems are represented; on the Y axis, average levels of each miRNA are represented. Levels of bta-miR-369-3p showed a skewed distribution, so they are shown using a box-and-whisker plot. The errors bars in the other plots indicate standard deviation.

**Figure 2:**
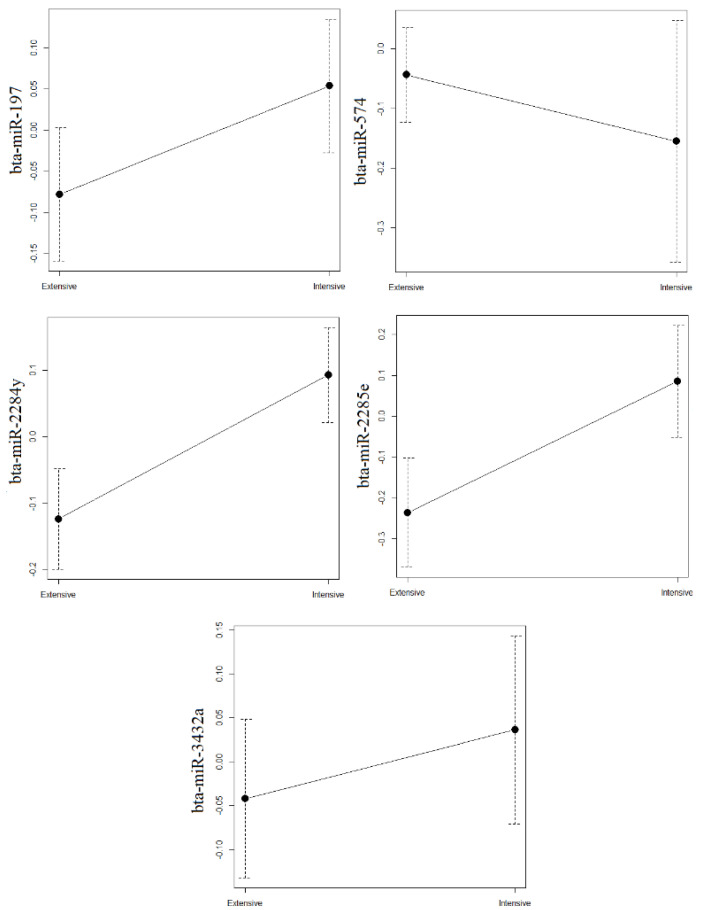
Average levels of miRNAs estimated by qRT-PCR in cellular fractions of milk from extensive and intensive farms. On the X axis, production systems are represented; on the Y axis, average levels of each miRNA are represented. The errors bars indicate standard deviation.

Based on analysis of the normalized miRNAs levels (Figure 1 and Figure 2), the only miRNA in the fat or cellular fraction that differed significantly between intensive and extensive production systems was bta-miR-215, in the fat fraction. This miRNA was significantly more abundant in the fat fraction of milk from intensive production (*p* = 0.030). The miRNAs bta-miR-2284y and bta-miR-2285e in the cellular fraction showed a trend towards lower levels in milk from extensive production, but the differences did not reach statistical significance.

### 3.3. Putative Target Gene and Pathway Analyses

Using Target Scan, 143 potential target genes were identified for bta-miR-215, which was abundant in intensive compared to extensive dairy production (Table S2). Among these targets, one gene was particularly involved in lipid metabolism and energy metabolism: the fatty acid binding protein 3 (Fabp3).

**Figure 3:**
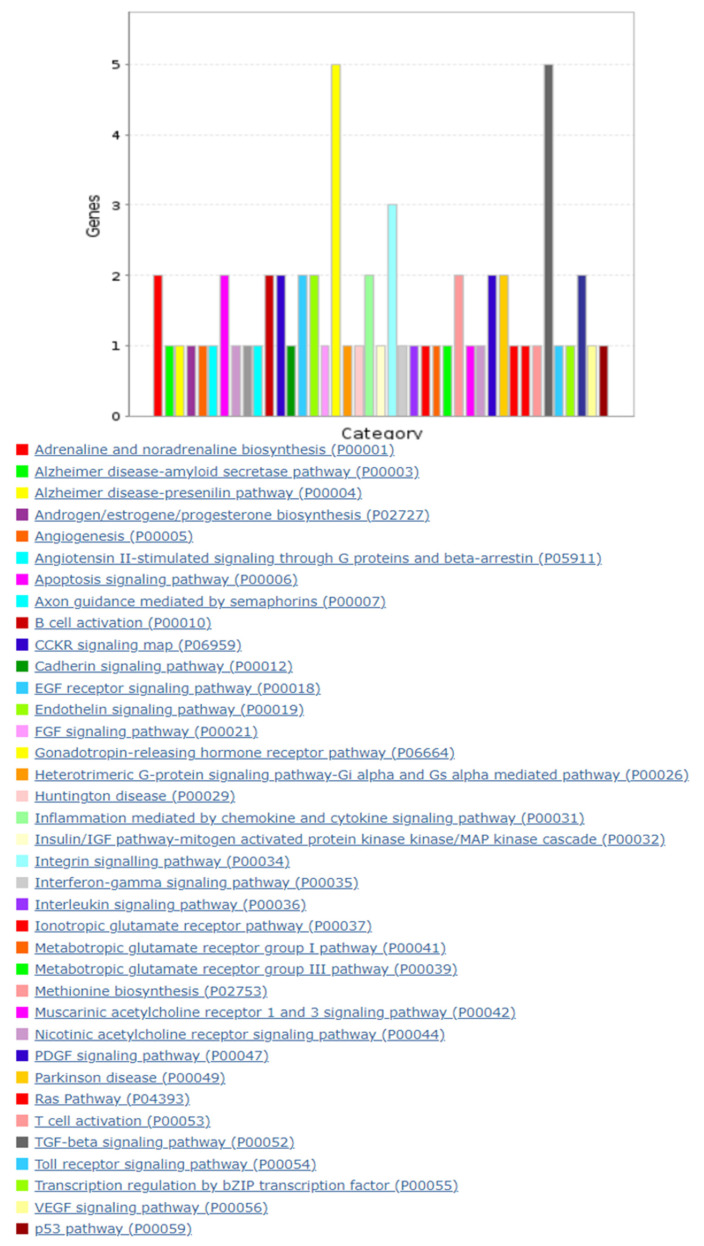
Functional Gene Ontology classification of bta-miR-215 pathway analysis of targeted genes.

The pathway analysis of bta-miR-215 target genes allowed for the identification of 41 associated biological pathways. The gonadotropin-releasing hormone receptor pathway and the Transforming Growth Factor β (TGF-β) signaling pathway (Figure 3) were highlighted, as they include the most bta-miR-215 target genes, i.e., Activin Receptor Type-2B (Acvr2b), Activin A Receptor, Type 2A (Acvr2a), Mitogen-Activated Protein Kinase 1 (Mapk1), and bone morphogenetic protein receptor type II (Bmpr2).

## 4. Discussion

The objective of this study was to identify a set of miRNAs in milk whose levels differed with the production system, in order to (1) determine whether miRNAs profiling can be used to authenticate milk from a given production system, and (2) investigate whether the production system can influence the functional properties of agri-food products such as bovine milk. To address these questions, we sampled milk from dairy farms in Asturias in northern Spain that applied intensive or extensive production practices, which differ significantly in numerous characteristics that can alter milk quality, including feeding management, animal density, and access to exercise through grazing [29].

The origin of miRNAs in milk is controversial, and most miRNAs in milk are not found in blood [30]. In addition, the miRNA profile in milk differs across the fractions of fat, whey, and cells [12]. In the present work, we decided not to study the whey fraction because its miRNA content is lower, its miRNA profile is highly similar to that of milk fat [12], and it is not exploited in certain dairy industry practices, such as cheese production.

We successfully isolated total RNA from fat and cellular fractions of bovine milk, especially considering that the samples came from milk tanks on commercial dairy farms. The RIN was low for RNA from milk fat, which likely reflects its abundant content of low-molecular-weight RNA, which is different from ribosomal RNA [12].

RNA sequencing analysis confirmed different RNA profiles in the fat and cellular fractions of bovine milk (Table 3). Interestingly, we did not identify significant differences in miRNAs levels between the two fractions, although we did obtain what appears to be the first evidence that miRNAs in the fat fraction differ between milk from intensive or extensive production systems. Given that milk has been proposed as a major epigenetic modulator of the gene expression of the milk recipient [31], and its modulation can be dependent on the amount of miRNAs [32], our results imply that the animal production system can influence the functional properties of agri-food products of animal origin.

Sequencing results did not allow for the identification of miRNAs that were specific to a given production system in either the fat or cellular fraction. Therefore, we focused on miRNAs whose levels differed between the two systems, and we validated a subset of the promising miRNAs using quantitative RT-PCR. The only miRNA that we validated to differ significantly between the two production systems was bta-miR-215 in the fat fraction (Table 5). This miRNA was upregulated in milk from intensive production. At the moment, we can only speculate as to why intensive production might upregulate this miRNA. One possibility is that its upregulation somehow compensates for poor efficiency of feed conversion into milk: dairy cows with medium potential show lower conversion efficiency during indoor feeding than during grazing in pastures [33], and Angus cows, less efficient at feed conversion, show upregulation of bta-miR-215 [34]. Consistent with this possibility is that heat and oxidative stress, which can easily occur in intensive production systems, upregulate bta-miR-215 in the serum of Holstein cows [35].

Among the 143 genes identified by bioinformatic tools, Fabp3 stands out for its involvement in fatty acid transport and activation in the bovine mammary gland [36]. Vargas-Bello-Perez et al. (2020) [37] demonstrated that when the diet of Holstein dairy cows is supplemented with hydrogenated vegetable oil, Fabp3 is downregulated in milk somatic cells. Likewise, when fed a high-concentrate diet, the fatty acid transporter Fabp3 is inhibited in mammary glands [38]. In our study, bta-miR-215 increased in milk in response to intensive farming conditions, which are generally associated with high-energy diets, which is consistent with its likely involvement in the downregulation of Fabp3. A direct relationship between bta-miR-215 and Fabp3 has, indeed, been validated in bone marrow mesenchymal stem cells [39].

Apart from Fabp3, another two genes are of particular interest: Mapk1 and Bmpr2. Mapk1 is involved in the regulation of milk protein synthesis [40], and Bmpr2 is associated with the glucose metabolism and insulin response. In fact, when Bmpr2 is altered, it likely blunts glucose response and lipid uptake [41]. Altogether, they likely have a negative impact on protein and lactose synthesis in animals bred under intensive dairy systems.

We also identified the miRNAs bta-mir-2284y and bta-mir-2285e in the cellular fraction of milk whose levels tended to differ between intensive and extensive production systems. Further analysis showed that these differences became significant when we compared animals on diets containing more or fewer than 10 kg of concentrates per day (data not shown). These results suggest that our failure to detect significant differences in levels of bta-mir-2284y and bta-mi-2285e in the overall analysis may reflect milk sampling from commercial farms, as well as the difficulty of defining intensive and extensive production. Further studies should examine these two miRNAs as additional potential biomarkers for authentication and functional analysis of agri-foods.

Finally, our study presents several limitations. The study was carried out in commercial farms, where managing conditions and diets are quite different even within the same group. Validation in controlled conditions at experimental farms, which reduce internal variance of the experimental groups, should help to identify miRNAs as putative biomarkers for dairy production systems. In vitro experiments will help to elucidate the functional features of milk produced under different dairy systems.

## 5. Conclusions

We investigated differences in miRNA profiles of raw cow tank milk from commercial farms which applied either intensive or extensive production systems. We identified bta-miRNA-215 in the fat fraction of milk as a possible biomarker of milk from intensive production systems. Our results imply that the type of production system can influence miRNA levels, and, therefore, functional properties of bovine milk, as well as, potentially, other agri-food products of animal origin.

## Figures and Tables

**Table 1:** Characteristics and diet of cows on the dairy farms where milk was sampled for RNA sequencing.

Production System	Number of Cows	Milk Production (L/Day/Cow)	Grazing (h/Day)	Grass Silage (kg F */Day/Cow)	Maize Silage (kg F */Day/cow)	Hay (kg F */Day/Cow)	Concentrate (kg F */Day/Cow)
Intensive (3 farms)	51	30	0	17	10.0	2	10
	65	28	0	10	15.0	10	11
	90	29	0	16	20.0	2	12
Extensive (3 farms)	24	21	20	10	0.0	2	7
	14	31	>12	14	0.0	6	6
	15	29	18	15	0.0	3	6

* kg F, kg of fresh matter.

**Table 2:** Characteristics and diet from twenty dairy farms featuring extensive or intensive production systems, where milk was sampled for quantitative real-time PCR validation.

Production System	Number of Cows	Milk Production (L/Day Cow)	Grazing (h/Day)	Grass Silage (kg F */Day/Cow)	Maize Silage (kg F */Day/Cow)	Hay (kg F */Day/Cow)	Concentrate (kg F */Day/Cow)
Intensive (10 farms)	124	37.4	0	8.0	30.0	0.8	11.5
	116	37.0	0	10.0	30.0	0.0	10.5
	90	29.0	0	16.0	20.0	3.0	12.0
	240	36.0	0	10.0	16.0	2.5	12.0
	250	38.0	0	12.0	30.0	0.9	12.3
	37	27.0	0	14.0	28.0	0.0	10.5
	110	30.0	0	16.0	20.0	0.0	11.0
	72	28.0	0	15.0	20.0	2.5	12.0
	118	36.0	0	16.0	22.0	0.0	10.5
	124	37.0	0	11.0	20.0	4.5	12.0
Extensive (10 farms)	20	21.0	6	10.0	0.0	6.8	6.5
	24	21.0	20	12.0	0.0	0.0	7.0
	12	26.2	22	0.0	0.0	6.0	4.0
	8	18.8	21	0.0	0.0	4.6	4.1
	35	19.5	20	14.0	0.0	0.0	4.7
	15	29.0	18	33.0	0.0	0.7	6.2
	30	27.0	18	0.0	0.0	4.0	8.0
	7	20.0	22	6.0	0.0	0.0	5.0
	16	23.0	21	0.0	0.0	0.0	5.0
	22	24.0	20	0.0	0.0	0.0	6.0

* kg F, kg of fresh matter.

**Table 3:** Read mapping statistics.

		Total Reads	Small RNA Reads	Small RNAs (%)						
				Total	rRNA	snRNA	snoRNA	tRNA	miRNA	ncRNA
**Cellular fraction**	Intensive Farms	32,560,190	16,693,358	0.5	12.7	3.3	5.7	45.0	2.2	31.1
		26,678,803	13,626,331	0.5	8.3	2.0	4.7	64.3	0.9	19.8
		23,616,528	11,871,084	0.5	16.5	3.7	5.5	37.6	2.0	34.8
	Extensive Farms	19,944,125	9,753,050	0.5	13.6	3.1	5.6	51.5	1.4	24.7
		21,166,161	9,650,109	0.5	14.9	3.9	6.6	45.4	1.4	27.8
		20,137,934	10,053,989	0.5	15.9	3.9	6.1	36.8	2.3	34.9
**Fat fraction**	Intensive Farms	7,620,977	3,867,636	0.5	20.6	3.4	6.1	29.8	1.7	38.4
		7,209,138	3,139,312	0.4	21.9	3.7	6.6	25.3	1.9	40.6
		6,180,232	3,115,110	0.5	26.0	3.8	6.0	15.8	1.6	46.9
	Extensive Farms	7,056,743	2,599,438	0.4	19.4	3.7	8.4	24.8	2.2	41.5
		6,682,264	3,279,814	0.5	25.0	4.1	7.1	16.9	2.4	44.5
		7,035,380	3,854,280	0.6	25.9	4.3	7.0	19.2	2.3	41.4

rRNA: ribosomal ribonucleic acid; snRNA: small nuclear RNA; snoRNA: small nucleolar RNA; tRNA: transfer RNA; miRNA: microRNA; ncRNA: non-coding RNA.

**Table 4:** Differences in abundance of small RNA classes depending on milk fraction and production system.

Small RNA Class	*p*-Value Based on Student’s *t* Test		
	Cellular vs. Fat Fraction *	Cellular Fraction: Extensive vs. Intensive	Fat Fraction: Extensive vs. Intensive
rRNA	0.004	0.513	0.827
snRNA	0.196	0.268	0.184
snoRNA	0.024	0.127	0.050
tRNA	0.004	0.827	0.513
miRNA	0.260	0.825	0.040
Non-coding RNA	0.004	0.827	0.513

* Both intensive and extensive production systems together. rRNA: ribosomal ribonucleic acid; snRNA: small nuclear RNA; snoRNA: small nucleolar RNA; tRNA: transfer RNA; miRNA: microRNA; ncRNA: non-coding RNA. In bold are significant differences.

**Table 5:** Ranking average of the first ten differentially expressed miRNAs in the fat fraction, the three applied tests’ values, and their rankings.

miRNA	Result for			Ranking According to			Average Ranking
	Test 1 ^a^	Test 2 ^b^	Test 3 ^c^	Test 1	Test 2	Test 3	
bta-miR-215 *	3.180	0.010	0.960	1	1	1	1.0
bta-miR-369-3p *	1.760	0.020	0.900	3	2	2	2.3
bta-miR-6520 *	1.360	0.030	0.850	4	3	4	3.7
bta-miR-7863 *	1.970	0.080	0.860	2	7	3	4.0
bta-miR-133a *	1.300	0.040	0.840	5	5	5	5.0
bta-miR-532	1.260	0.040	0.840	6	4	6	5.3
bta-miR-148a	1.210	0.120	0.780	7	13	7	9.0
bta-miR-138	1.000	0.070	0.770	22	6	8	12.0
bta-miR-450a	1.190	0.140	0.760	8	18	10	12.0
bta-miR-6527	1.010	0.090	0.770	21	8	9	12.7

* These miRNAs were validated using quantitative RT-PCR. ^a^ Strict Cohen’s d test. ^b^ Student’s *t* test. ^c^ Correlation coefficient test.

**Table 6:** Ranking average of the first ten differentially expressed miRNAs in the cellular fraction, the three applied tests’ values, and their rankings.

miRNA	Result for			Ranking According to			Average Ranking
	Test 1 ^a^	Test 2 ^b^	Test 3 ^c^	Test 1	Test 2	Test 3	
bta-miR-574 *	5.770	0.000	0.990	1	1	1	1.0
bta-miR-3432a *	5.520	0.010	0.980	2	3	2	2.3
bta-miR-2285e *	2.540	0.010	0.950	5	2	3	3.3
bta-miR-197 *	1.970	0.010	0.920	6	4	5	5.0
bta-miR-2284y *	2.750	0.020	0.940	3	8	4	5.0
bta-miR-219	1.740	0.010	0.910	9	5	7	7.0
bta-miR-2397-3p	1.770	0.020	0.900	8	7	8	7.7
bta-miR-2308	2.560	0.050	0.910	4	14	6	8.0
bta-miR-2419-5p	1.620	0.020	0.890	11	6	9	8.7
bta-miR-2409	1.790	0.040	0.890	7	12	10	9.7

* These miRNAs were validated using quantitative RT-PCR. ^a^ Strict Cohen’s d test. ^b^ Student’s *t* test. ^c^ Correlation coefficient test.

**Table 7:** Ranking of the most stable miRNAs according to the coefficient of variation.

Milk Fraction	miRNA	Coefficient of Variation
Fat	bta-miR-532	0.060
	bta-miR-151-3p	0.070
	bta-miR-27b	0.090
	bta-miR-103	0.090
	bta-miR-30a-5p	0.090
	bta-miR-99a-3p	0.090
Cellular	bta-miR-103	0.080
	bta-miR-107	0.090
	bta-miR-181a	0.090
	bta-miR-28	0.100
	bta-miR-345-3p	0.100
	bta-miR-28342	0.100

## Data Availability

Not applicable.

## Supplementary Materials

The following supporting information can be downloaded at: https://www.mdpi.com/article/10.3390/vetsci9120661/s1, Table S1: Sequencing RPM; Table S2: Selection of Predicted *bta-miR-215* Targets from Target Scan database.
