# Supplementary material for: Differences in the microRNAs Levels of Raw Milk from Dairy Cattle Raised under Extensive or Intensive Production Systems
Source: Vet Sci. 2022 Nov 27;9(12):661. doi: 10.3390/vetsci9120661 (PMC9783024; doi:10.3390/vetsci9120661)
Supplement: Supplementary file 1 [file vetsci-09-00661-s001.zip › SuppTable2-def.pdf]

Supplementary Table S2: Selection of Predicted *bta-miR-215* Targets from Target Scan database

Ortholog of target gene	Representative transcript	Gene name
ACVR2A	ENST0000024 1416.7	activin A receptor, type IIA
ACVR2B	ENST0000035 2511.4	activin A receptor, type IIB
AL354993.1	ENST0000043 9873.2	Cell growth-inhibiting protein 7; HCG1784586; Uncharacterized protein
ALCAM	ENST0000030 6107.5	activated leukocyte cell adhesion molecule
ALG6	ENST0000037 1108.4	ALG6, alpha-1,3-glucosyltransferase
ALX1	ENST0000031 6824.3	ALX homeobox 1
AMER1	ENST0000033 0258.3	APC membrane recruitment protein 1
ANAPC16	ENST0000029 9381.4	anaphase promoting complex subunit 16
ANKRD32	ENST0000026 5140.5	ankyrin repeat domain 32
ANKRD44	ENST0000028 2272.8	ankyrin repeat domain 44
APTX	ENST0000043 6040.2	aprataxin
ARFGEF1	ENST0000026 2215.3	ADP-ribosylation factor guanine nucleotide-exchange factor 1 (brefeldin A-inhibited)
ARHGEF39	ENST0000034 3259.3	Rho guanine nucleotide exchange factor (GEF) 39
ARL4C	ENST0000039 0645.2	ADP-ribosylation factor-like 4C
ASB6	ENST0000027 7459.4	ankyrin repeat and SOCS box containing 6
ATP6V1C2	ENST0000038 1661.3	ATPase, H+ transporting, lysosomal 42kDa, V1 subunit C2
B3GALNT2	ENST0000036 6600.3	beta-1,3-N-acetylgalactosaminyltransferase 2
BHLHE22	ENST0000032 1870.1	basic helix-loop-helix family, member e22
BLCAP	ENST0000039 7137.1	bladder cancer associated protein
BMPR2	ENST0000037 4574.2	bone morphogenetic protein receptor, type II (serine/threonine kinase)
BNC2	ENST0000038 0672.4	basonuclin 2
BRD3	ENST0000030 3407.7	bromodomain containing 3

C1orf21	ENST0000023 5307.6	chromosome 1 open reading frame 21
C5orf27	ENST0000043 6592.1	chromosome 5 open reading frame 27
CBL	ENST0000026 4033.4	Cbl proto-oncogene, E3 ubiquitin protein ligase
CCDC171	ENST0000038 0701.3	coiled-coil domain containing 171
CCNT2	ENST0000029 5238.6	cyclin T2
CDC42SE2	ENST0000050 5065.1	CDC42 small effector 2
CDC6	ENST0000020 9728.4	cell division cycle 6
CDON	ENST0000039 2693.3	cell adhesion associated, oncogene regulated
CLSTN1	ENST0000037 7298.4	calsyntenin 1
CLVS2	ENST0000027 5162.5	clavesin 2
CNKSR3	ENST0000060 7772.1	CNKSR family member 3
COL5A1	ENST0000037 1817.3	collagen, type V, alpha 1
CPEB4	ENST0000026 5085.5	cytoplasmic polyadenylation element binding protein 4
CREB5	ENST0000035 7727.2	cAMP responsive element binding protein 5
CTCF	ENST0000026 4010.4	CCCTC-binding factor (zinc finger protein)
CTH	ENST0000041 1986.2	cystathionase (cystathionine gamma-lyase)
CXCR5	ENST0000029 2174.4	chemokine (C-X-C motif) receptor 5
DBT	ENST0000037 0132.4	dihydrolipoamide branched chain transacylase E2
DCC	ENST0000044 2544.2	deleted in colorectal carcinoma
DDX49	ENST0000043 8170.2	DEAD (Asp-Glu-Ala-Asp) box polypeptide 49
DDX50	ENST0000037 3585.3	DEAD (Asp-Glu-Ala-Asp) box polypeptide 50
DICER1	ENST0000054 1352.1	dicer 1, ribonuclease type III
DLG5	ENST0000037 2391.2	discs, large homolog 5 (Drosophila)
DNAH17-AS1	ENST0000059 8378.1	DNAH17 antisense RNA 1

DNAJC19	ENST0000048 6355.1	DnaJ (Hsp40) homolog, subfamily C, member 19
DYRK3	ENST0000036 7108.3	dual-specificity tyrosine-(Y)-phosphorylation regulated kinase 3
EIF1	ENST0000046 9257.1	eukaryotic translation initiation factor 1
EIF5A2	ENST0000047 4096.1	eukaryotic translation initiation factor 5A2
EMC7	ENST0000025 6545.4	ER membrane protein complex subunit 7
ENC1	ENST0000030 2351.4	ectodermal-neural cortex 1 (with BTB domain)
ENY2	ENST0000052 0147.1	enhancer of yellow 2 homolog (Drosophila)
FABP3	ENST0000037 3713.2	fatty acid binding protein 3, muscle and heart (mammary-derived growth inhibitor)
FNDC3B	ENST0000033 6824.4	fibronectin type III domain containing 3B
FOXN1	ENST0000022 6247.2	forkhead box N1
FRK	ENST0000060 6080.1	fyn-related kinase
FTO	ENST0000047 1389.1	fat mass and obesity associated
FUNDC2	ENST0000036 9498.3	FUN14 domain containing 2
GABPB2	ENST0000036 8918.3	GA binding protein transcription factor, beta subunit 2
GALNTL6	ENST0000050 6823.1	UDP-N-acetyl-alpha-D-galactosamine:polypeptide N-acetylgalactosaminyltransferase-like 6
GLP1R	ENST0000037 3256.4	glucagon-like peptide 1 receptor
GPAM	ENST0000034 8367.4	glycerol-3-phosphate acyltransferase, mitochondrial
GPR111	ENST0000039 8742.2	G protein-coupled receptor 111
GPR126	ENST0000023 0173.6	G protein-coupled receptor 126
GPR22	ENST0000030 4402.4	G protein-coupled receptor 22
GRIN2B	ENST0000060 9686.1	glutamate receptor, ionotropic, N-methyl D-aspartate 2B
HIGD1A	ENST0000032 1331.7	HIG1 hypoxia inducible domain family, member 1A
HOXA11	ENST0000000 6015.3	homeobox A11
HS6ST1	ENST0000025 9241.6	heparan sulfate 6-O-sulfotransferase 1

IGDCC3	ENST0000032 7987.4	immunoglobulin superfamily, DCC subclass, member 3
IL17RD	ENST0000029 6318.7	interleukin 17 receptor D
KCNA7	ENST0000022 1444.1	potassium voltage-gated channel, shaker-related subfamily, member 7
KCNK1	ENST0000036 6621.3	potassium channel, subfamily K, member 1
KIF1B	ENST0000037 7086.1	kinesin family member 1B
KLHL15	ENST0000032 8046.8	kelch-like family member 15
KPNA4	ENST0000033 4256.4	karyopherin alpha 4 (importin alpha 3)
KPNA6	ENST0000037 3625.3	karyopherin alpha 6 (importin alpha 7)
LDB3	ENST0000054 2786.1	LIM domain binding 3
LMTK2	ENST0000029 7293.5	lemur tyrosine kinase 2
LPAR4	ENST0000043 5339.3	lysophosphatidic acid receptor 4
LRIG2	ENST0000036 1127.5	leucine-rich repeats and immunoglobulin-like domains 2
MAPK1	ENST0000021 5832.6	mitogen-activated protein kinase 1
MFAP3	ENST0000043 6816.1	microfibrillar-associated protein 3
MIER3	ENST0000038 1226.3	mesoderm induction early response 1, family member 3
MMP16	ENST0000028 6614.6	matrix metallopeptidase 16 (membrane-inserted)
MSN	ENST0000036 0270.5	moesin
MTMR4	ENST0000057 9925.1	myotubularin related protein 4
MYO9A	ENST0000056 4571.1	myosin IXA
NAA50	ENST0000024 0922.3	N(alpha)-acetyltransferase 50, NatE catalytic subunit
NDUFB9	ENST0000027 6689.3	NADH dehydrogenase (ubiquinone) 1 beta subcomplex, 9, 22kDa
NFAT5	ENST0000035 4436.2	nuclear factor of activated T-cells 5, tonicity-responsive
NIPBL	ENST0000044 8238.2	Nipped-B homolog (Drosophila)
NKX2-5	ENST0000042 4406.2	NK2 homeobox 5

NR6A1	ENST0000048 7099.2	nuclear receptor subfamily 6, group A, member 1
PAPLN	ENST0000038 1166.3	papilin, proteoglycan-like sulfated glycoprotein
PCDH17	ENST0000037 7918.3	protocadherin 17
PCK2	ENST0000055 9250.1	phosphoenolpyruvate carboxykinase 2 (mitochondrial)
PHTF2	ENST0000041 6283.2	putative homeodomain transcription factor 2
PLXNB2	ENST0000044 9103.1	plexin B2
PMAIP1	ENST0000031 6660.6	phorbol-12-myristate-13-acetate-induced protein 1
PRKG1	ENST0000037 3985.1	protein kinase, cGMP-dependent, type I
PTPRT	ENST0000037 3187.1	protein tyrosine phosphatase, receptor type, T
RAD51L3-RFFL	ENST0000059 3039.1	Uncharacterized protein
RAD54B	ENST0000029 7592.5	RAD54 homolog B (S. cerevisiae)
RAP1GAP2	ENST0000025 4695.8	RAP1 GTPase activating protein 2
RFFL	ENST0000031 5249.7	ring finger and FYVE-like domain containing E3 ubiquitin protein ligase
RICTOR	ENST0000035 7387.3	RPTOR independent companion of MTOR, complex 2
RUNX1	ENST0000034 4691.4	runt-related transcription factor 1
RUNX1T1	ENST0000052 3629.1	runt-related transcription factor 1; translocated to, 1 (cyclin D-related)
SCARF1	ENST0000057 1272.1	scavenger receptor class F, member 1
SCN1A	ENST0000042 3058.2	sodium channel, voltage-gated, type I, alpha subunit
SLC39A6	ENST0000026 9187.5	solute carrier family 39 (zinc transporter), member 6
SLC9B2	ENST0000050 3103.1	solute carrier family 9, subfamily B (NHA2, cation proton antiporter 2), member 2
SNX33	ENST0000030 8527.5	sorting nexin 33
SOAT1	ENST0000036 7619.3	sterol O-acyltransferase 1
SOGA3	ENST0000055 6132.1	SOGA family member 3
SRGAP3	ENST0000038 3836.3	SLIT-ROBO Rho GTPase activating protein 3

SRPX2	ENST0000037 3004.3	sushi-repeat containing protein, X-linked 2
SRSF6	ENST0000024 4020.3	serine/arginine-rich splicing factor 6
TAOK1	ENST0000026 1716.3	TAO kinase 1
TRERF1	ENST0000054 1110.1	transcriptional regulating factor 1
TRIM44	ENST0000029 9413.5	tripartite motif containing 44
TRPM7	ENST0000056 0955.1	transient receptor potential cation channel, subfamily M, member 7
UBE2QL1	ENST0000039 9816.3	ubiquitin-conjugating enzyme E2Q family-like 1
UBE2V2	ENST0000052 3111.2	ubiquitin-conjugating enzyme E2 variant 2
VPS33A	ENST0000026 7199.4	vacuolar protein sorting 33 homolog A (S. cerevisiae)
VTI1B	ENST0000055 4659.1	vesicle transport through interaction with t-SNAREs 1B
WNK1	ENST0000031 5939.6	WNK lysine deficient protein kinase 1
WSCD2	ENST0000033 2082.4	WSC domain containing 2
WWC2	ENST0000040 3733.3	WW and C2 domain containing 2
XIAP	ENST0000037 1199.3	X-linked inhibitor of apoptosis
YY1	ENST0000026 2238.4	YY1 transcription factor
ZBTB18	ENST0000035 8704.4	zinc finger and BTB domain containing 18
ZBTB34	ENST0000031 9119.4	zinc finger and BTB domain containing 34
ZC3HAV1	ENST0000024 2351.5	zinc finger CCCH-type, antiviral 1
ZEB2	ENST0000055 8170.2	zinc finger E-box binding homeobox 2
ZFHX3	ENST0000026 8489.5	zinc finger homeobox 3
ZFP36L1	ENST0000055 5997.1	ZFP36 ring finger protein-like 1
ZNF280C	ENST0000037 0978.4	zinc finger protein 280C
ZNF536	ENST0000035 5537.3	zinc finger protein 536
ZPBP2	ENST0000037 7940.3	zona pellucida binding protein 2

ZRANB3	ENST00000401392.1	zinc finger, RAN-binding domain containing 3
